# Advancements in Electronic Medical Records for Clinical Trials: Enhancing Data Management and Research Efficiency

**DOI:** 10.3390/cancers17091552

**Published:** 2025-05-02

**Authors:** Mingyu Lee, Kyuri Kim, Yoojin Shin, Yoonji Lee, Tae-Jung Kim

**Affiliations:** 1 College of Medicine, The Catholic University of Korea, 222 Banpo-daero, Seocho-gu, Seoul 06591, Republic of Korea; mkl0107@catholic.ac.kr (M.L.); yoojinshin@catholic.ac.kr (Y.S.); gina228@catholic.ac.kr (Y.L.); 2 College of Medicine, Ewha Womans University, 25 Magokdong-ro 2-gil, Gangseo-gu, Seoul 03760, Republic of Korea; kkyuri01@ewhain.net; 3 Department of Hospital Pathology, Yeouido St. Mary’s Hospital, College of Medicine, The Catholic University of Korea, 10, 63-ro, Yeongdeungpo-gu, Seoul 07345, Republic of Korea

**Keywords:** electronic medical record, clinical trial, privacy, artificial intelligence, regulation, automation

## Abstract

Recent advancements in electronic medical records (EMRs) have transformed clinical trials and healthcare systems by improving data accuracy, regulatory compliance, and integration with decision support tools. These innovations enhance trial efficiency, streamline patient recruitment, and enable large-scale data analysis while bridging clinical practice with research. Despite these benefits, challenges such as data standardization, privacy concerns, and usability issues persist. Overcoming these barriers through policy reforms, technological innovations, and robust methodologies is essential to maximizing the potential of EMRs. We examine current developments, challenges, and future directions for optimizing EMRs in clinical trials and healthcare delivery.

## Simple Summary

Electronic medical records (EMRs) are increasingly integral to modern clinical trials, offering enhanced data accuracy, efficient participant recruitment, and seamless integration of real-world data. Their evolution from basic recordkeeping to advanced, AI-supported research tools has enabled improved trial design, decision support, and operational efficiency. Despite these advancements, key challenges persist—including issues with data standardization, privacy, interoperability, and limited stakeholder coordination. Addressing these barriers through policy reform, technological innovation, and collaborative infrastructure development is essential for maximizing the impact of EMRs on clinical research and healthcare delivery.

## 1. Introduction

The integration of electronic medical records (EMRs) has transformed healthcare by enabling real-time access to patient data, supporting clinical decision making, and improving care delivery efficiency. While EMRs were originally developed to digitize medical documentation, their functionality has expanded significantly to include roles in research, including clinical trials.

Among these applications, clinical trials have particularly benefited from EMR adoption. EMRs facilitate automated data collection, reduce manual errors, improve regulatory compliance, and enhance patient recruitment and follow-up. Moreover, their integration with artificial intelligence (AI), machine learning (ML), and natural language processing (NLP) has enabled advanced data analytics and decision support capabilities that hold significant promise for modern clinical research. Despite these advancements, technical and ethical challenges persist, especially regarding data interoperability, privacy, standardization, and system usability across diverse settings.

Given the increasing reliance on EMRs in research environments, this review aims to critically examine their evolving role in clinical trials. It explores the technological and organizational drivers of EMR integration, evaluates barriers to implementation, and analyzes emerging frameworks for optimizing EMR utility in clinical research. Special attention is given to regulatory considerations, sponsor involvement, and system interoperability. By addressing these dimensions, we aim to answer the following research question: How can electronic medical records be optimized to improve the efficiency, reliability, and scalability of clinical trials while addressing current technical and ethical challenges?

## 2. From Recordkeeping to Research: The Expanding Role of EMRs

### 2.1. From Paper to Digital: The Evolution of Medical Records

The origins of medical recordkeeping go back to antiquity, where early documentat- ions–such as Corpus Hippocraticum–captured diagnostic and therapeutic practices primarily for educational use [1,2,3]. However, consistent and structured medical records were rare throughout ancient and medieval times, and their purpose varied across institutions and regions [4,5].

Significant transformation began during the Enlightenment and into the 19th century, as hospitals in Europe and the United States shifted from religious shelters to centers of scientific medicine [6,7,8]. Physicians began to record clinical observations systematically, and hospitals like Hôtel-Dieu in Paris and Charité in Berlin pioneered patient registries and daily medical documentation [9,10,11,12]. These changes laid the groundwork for using records not only in care but also in medical education and research [13,14,15].

By the early 20th century, efforts to standardize medical records emerged. Innovations such as assigning unique patient identifiers and consolidating fragmented documentation—championed by figures like Henry Plummer—marked the transition toward structured, patient-centered records [14,16]. Institutions like the Mayo Clinic became models for organizing and managing clinical data [5,17]. During this period, national organizations such as the American College of Surgeons initiated campaigns to formalize medical documentation practices across hospitals [12,18].

Although these paper-based systems lacked many of the efficiencies of today’s digital tools, they established core principles—standardization, longitudinal tracking, and data reuse—that would later inform the design of EMRs [5,10]. Understanding these historical underpinnings helps contextualize current challenges and opportunities in using EMRs to support clinical trials.

This historical foundation illustrates how the core functions of recordkeeping, documentation, continuity of care, and data reusability—set the stage for digitization efforts in the late 20th century. The emergence of EMRs represented not just a technological innovation but a paradigm shift in how clinical information was captured, stored, and utilized.

**Figure 1:**
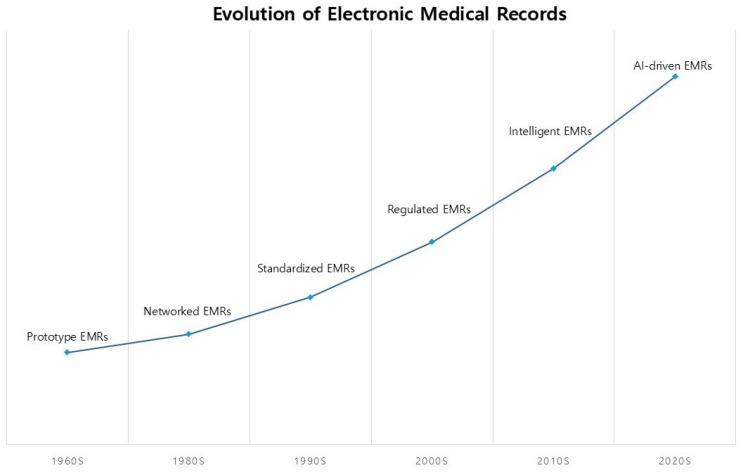
Evolution of electronic medical records.

The chronological evolution of electronic medical records is illustrated in Figure 1.

### 2.2. Infrastructure and Policy Factors in EMR Adoption

The concept of EMRs first emerged in the 1960s, with early systems like the Problem-Oriented Medical Information System and the Regenstrief Institute’s EMR prototype aiming to digitize patient data [5,19]. However, adoption remained limited for decades due to high implementation costs, limited infrastructure, and clinician resistance [20,21]. By the 1990s, EMRs supported basic functions like lab result documentation, scheduling, and billing, but were not widely adopted in research settings due to concerns over usability and efficiency [22,23].

As internet access expanded in the late 1990s and early 2000s, EMRs evolved into web-based systems that enabled real-time access to clinical data such as lab reports and physician notes [24,25]. Technical standards like HL7 and DICOM (Digital Imaging and Communications in Medicine) helped improve interoperability, but issues around data fragmentation, ownership, and quality remained [26,27].

In addition to communication protocols like HL7, several standard frameworks have been pivotal in structuring and supporting EMR development. Business Process Model and Notation (BPMN) is widely used to visualize and optimize clinical workflows, aiding in the alignment of healthcare processes with digital recordkeeping systems. For example, BPMN has been successfully applied to analyze time-variant clinical pathways using event log data extracted from EMRs, enabling detailed variance analysis in real-world healthcare delivery [28]. Unified Modeling Language (UML) offers a standardized method for designing EMR software architecture, enhancing modularity and clarity during system development [29]. For medical imaging, the DICOM standard plays a critical role by ensuring the consistent storage, transmission, and interpretation of diagnostic images across healthcare systems. Importantly, anonymization frameworks for DICOM data have been developed to support privacy-preserving secondary use in research, particularly in sensitive domains like radiation therapy [30]. Together, these standards form a foundational layer for achieving interoperability, system scalability, and effective integration of EMRs in complex medical environments [31].

However, despite the existence of these international standards, their implementation in real-world EMR systems remains inconsistent. The inconsistent adoption of established modeling and communication standards continues to hinder EMR interoperability across institutions. Standards such as BPMN, UML, and DICOM have been proposed to improve structured data capture and system integration. However, their implementation remains fragmented. A lack of unified functional reference models leads to variability in EMR system capabilities, limiting the consistent application of such standards across vendors and countries. This impairs cross-platform data exchange and obstructs efforts to standardize clinical workflows. Moreover, although anonymization protocols for DICOM data exist, inconsistent adoption can lead to compliance challenges in multicenter research settings. Broader institutional alignment with international standards is essential to ensure reliable data management and secure information exchange across the clinical trial lifecycle [28,29,30,31].

However, despite these promising developments, the integration of clinical trial data into EMR systems remains limited. Sponsors, such as pharmaceutical companies, often retain data ownership, restricting access for providers and EMR vendors. This limits the incorporation of trial results into patient records and reduces their real-world impact. Additionally, the financial burden of data standardization and interoperability often falls on hospitals rather than sponsors, creating further disparities. While some countries have pursued public–private initiatives to address this gap, consistent access and data-sharing frameworks remain elusive.

The structure of a country’s healthcare system has played a crucial role in shaping the development, implementation, and utilization of EMRs. In the United States, where a mixed Bismarck-style model dominates, both public and private healthcare organizations have developed distinct EMR infrastructures based on their institutional contexts. For instance, Kaiser Permanente, a large nonprofit integrated delivery system, implemented an enterprise-wide EMR based on EpicCare and has used it extensively for clinical care and research [32,33]. This centralized approach has supported advanced functionalities such as cohort identification for rare diseases, as demonstrated in its study on membranous nephropathy using longitudinal EMR data [34].

Conversely, the U.S. Department of Veterans Affairs (VA), as a public provider, developed and maintained its own EMR system—Veterans Health Information Systems and Technology Architecture (VistA) [35]. This internally built platform emphasizes flexibility and long-term customization across different VA sites. Its structure supports scalable data extraction and has enabled predictive modeling in areas such as the early identification of systemic sclerosis complications [36].

Internationally, countries under the Beveridge model—such as the United Kingdom—have favored nationalized EMR systems. The NHS, for example, sought to deploy a unified national health IT system, though its implementation faced persistent technical and organizational setbacks, including insufficient training, user resistance, and limited system flexibility [37,38]. Even in centralized settings, clinician engagement and workflow alignment remain critical bottlenecks. On the other hand, Bismarck-model countries like Germany, which rely on decentralized payer–provider relationships, have struggled to standardize EMR platforms across institutions due to fragmented infrastructure, governance complexity, and heterogeneous digital readiness [37,39,40]. A lack of enforceable interoperability standards and inconsistent policy frameworks across stakeholders further complicate system integration.

These variations underscore the fact that EMR systems cannot be separated from the organizational and financial architecture of healthcare delivery. Tailoring EMR design and rollout strategies to match national health system models is critical to their success.

### 2.3. Institutional Use and System-Level Applications of EMRs

Following the HITECH Act in 2009, the adoption of EMRs in the United States accelerated, with more than half of physicians using electronic systems by 2011 [41,42]. Despite this growth, concerns about usability, physician burden, and clinical impact persisted [43,44]. EMRs were often criticized as less intuitive and more expensive compared to digital tools in other sectors, raising questions about their real-world value [45].

As digital infrastructure matured, EMRs began to support a wide range of functionalities, including clinical decision making, telemedicine, behavioral health integration, and mobile-based documentation [46,47,48,49]. These innovations laid the groundwork for system-level reforms such as integrated and bundled care.

EMRs play a foundational role in integrated care models, which aim to reduce care fragmentation and promote coordination across providers. In the UK and the Netherlands, shared access to EMRs has enabled smoother transitions between primary and secondary care and enhanced collaboration between hospitals and community services [50,51]. Similarly, bundled care—grouping related health conditions for optimized management—has been facilitated by EMR data workflows. For instance, data-driven models have used inpatient EMRs to identify phenotypic clusters, supporting the design of team-based care strategies [32,52].

These system-level applications of EMRs—particularly in coordinated and bundled care—have indirect implications for clinical trials. They provide infrastructure for longitudinal patient tracking, improve real-world data quality, and support patient-centered outcomes research. However, their success depends on leadership, stakeholder engagement, and effective governance [53,54].

As EMRs expand into non-hospital settings such as nursing homes and correctional facilities, issues of interoperability and equity emerge. Similarly, personal health records (PHRs), often integrated with EMRs, are gaining traction as tools for patient engagement, though barriers in access and legal frameworks remain [55,56]. Addressing these disparities is critical for ensuring representative data collection in research contexts.

Despite broad adoption, EMRs still face criticisms regarding clinician workload, data duplication, and limited flexibility. These challenges have led to the emergence of workarounds, medical scribes, and open source EMR platforms in low-resource settings [57,58]. Efforts to improve usability through design ergonomics and simulation-based training are ongoing [59,60].

In parallel, emerging technologies—including standardized APIs, decision support plug-ins, and potential genomic data integration—are being explored to extend EMR utility [61,62,63]. These advances may enable scalable support for adaptive clinical trials and precision medicine in the near future.

### 2.4. Decision Support and EMR-Enabled Clinical Trial Interventions

While the adoption of EMRs has expanded globally—with over 80% of hospitals and physician offices in the U.S. implementing EMR systems by 2021—their clinical impact remains mixed. Barriers such as unintuitive interfaces, inconsistent data quality, and clinician burnout continue to limit perceived value, despite high implementation rates [64].

Among their most promising applications, EMR-based clinical decision support (CDS) systems have gained attention for enhancing diagnostic accuracy, improving adherence to treatment guidelines, and issuing alerts for drug interactions and contraindications [65]. These tools improve patient safety and reduce medical errors, making them essential for both routine clinical care and research settings.

In the context of clinical trials, EMRs have shown tangible utility in enabling automated, guideline-based interventions. A study across three hospitals affiliated with the University of Pennsylvania demonstrated how EMR-integrated “nudge” interventions increased the rate of comprehensive molecular testing from 88% to 100%, and improved adherence to NCCN-concordant care from 78.2% to 89.8%. Additionally, the average time to obtain test results was reduced from 22 to 17 days. Importantly, similar gains were also observed in community hospitals, suggesting that EMR-based trial interventions can be scaled beyond academic centers.

These findings highlight how EMRs can bridge evidence-to-practice gaps, support trial implementation, and enhance data fidelity. However, significant limitations remain. The lack of standardized molecular testing reports and varying levels of insurance coverage pose barriers to broader application. Without interoperable standards, the potential of EMRs to harmonize trial data and streamline workflows will remain underutilized [66].

Pandemic-era responses further revealed EMRs’ potential to support real-time risk stratification and resource allocation. During COVID-19, EMRs were used to identify comorbidities, guide prognostic evaluation, and improve care coordination under diagnostic uncertainty. These applications demonstrate how EMRs can contribute to adaptive trial design and rapid-cycle analytics when properly integrated.

Ultimately, EMRs hold the infrastructure to support prospective trial interventions, real-time monitoring, and precision medicine. Yet, to realize this potential, greater investment in system standardization, vendor cooperation, and supportive policy frameworks is necessary.

## 3. Strategic Integration of EMRs into Clinical Trials

### 3.1. Digital Transformation of Clinical Trial Data Management

Clinical data management (CDM) is a foundational component of clinical trials, ensuring the collection, organization, and validation of data to uphold study integrity and regulatory compliance. Traditionally, CDM relied on manual processes, particularly paper-based case report forms (CRFs), which introduced substantial inefficiencies in large-scale or multicenter trials. In these early trials, patient data were manually recorded and physically transferred to central coordination centers. This process often led to delays in data entry, difficulties in monitoring data quality, and increased risks to data security. Studies have shown that paper-based reporting negatively impacted response rates from research centers and impeded timely trial execution. These operational bottlenecks underscored the need for digital transformation in clinical data workflows. The increasing scale and complexity of clinical trials rendered manual data entry unsustainable, catalyzing the development of electronic systems for data capture, validation, and monitoring. This shift set the stage for integrating EMRs into research infrastructures, bridging clinical care and trial data environments. By addressing the limitations of traditional CDM, early electronic systems laid a technical foundation for the modern use of EMRs in research—enabling greater efficiency, data accuracy, and real-time accessibility across trial sites.

### 3.2. Regulatory Framework and Recruitment Innovation with EMRs

The Common Rule, first published in 1991 and revised in 2018, provides the federal framework for protecting human subjects in clinical research. Key provisions include Institutional Review Board (IRB) oversight, informed consent requirements, and safeguards for participant privacy. The 2018 amendments specifically addressed digital-era concerns by clarifying the distinction between personal and personally identifiable information, and promoting the use of unique study identifiers to maintain participant anonymity [67].

Traditionally, clinical trial records were recorded on paper and manually transcribed into sponsor-managed databases using deidentified screening numbers. However, the 2018 Common Rule revision explicitly confirmed that electronically identifiable data can be maintained in secure electronic formats by research institutions, thus enabling more efficient data workflows while maintaining privacy protections.

As clinical trials have become increasingly complex and costly, electronic systems have emerged as essential tools for improving both research administration and direct participant care. These systems support protocol management, regulatory compliance, and real-time communication across research teams [67].

A central challenge in clinical trials is participant recruitment. Historically reliant on physician referrals, traditional recruitment approaches often introduce bias and logistical delays. In response, manual chart review using EMRs has become a standard method for identifying eligible candidates. However, this process remains labor-intensive and time-consuming.

To address this, several initiatives now leverage EMR data more systematically. For example, the TransCelerate e-Source initiative, launched in 2016, aims to enhance the integration of electronic data resources—including EMRs—into clinical trial design and execution. The initiative supports modernized enrollment workflows, facilitates site selection, and helps streamline eligibility screening using structured data and advanced informatics tools.

By aligning with privacy regulations and harnessing structured EMR data, these evolving recruitment strategies demonstrate the potential to improve trial efficiency, reduce bias, and increase enrollment rates—while maintaining compliance with ethical and regulatory standards.

**Figure 2:**
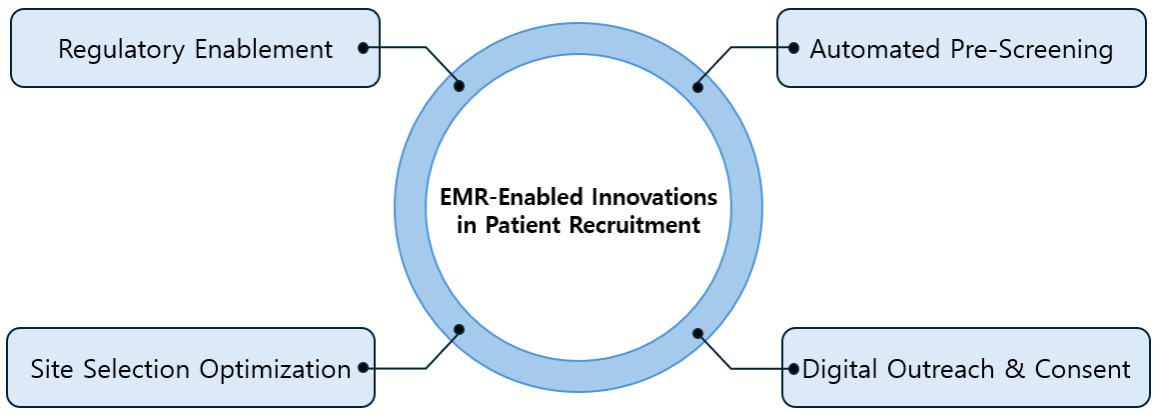
EMR-enabled innovation in patient recruitment.

The core innovations in EMR-facilitated patient recruitment strategies are summarized in Figure 2.

### 3.3. Functional Expansion of EMRs in Clinical Research

EMRs have become instrumental in bridging clinical research with real-world medical practice. Health technology companies increasingly utilize EMRs to compile patient demographic data, treatment histories, clinical outcomes, and genetic profiles. These datasets enable large-scale retrospective studies, support the development of machine learning models, and facilitate the evaluation of disease patterns and therapeutic responses [65].

In clinical trials, EMRs enhance data integrity by centralizing participant information such as medical histories and laboratory findings. Their integration reduces manual data entry errors, ensures consistent data formatting, and improves regulatory compliance. Furthermore, EMRs facilitate the separation of personally identifiable information from research records, allowing for secure data environments with differentiated access controls.

Clinical Trial Management Systems (CTMS) serve as core infrastructure for managing study-specific workflows—tracking participant enrollment, scheduling study visits, and overseeing compliance. When integrated with EMRs, CTMS platforms support real-time data sharing and bidirectional communication between clinical and research teams. These systems can automatically synchronize patient status updates, eligibility flags, and trial outcomes, thereby streamlining trial coordination and reducing administrative workload [67].

EMRs also support pre-screening and recruitment by enabling structured queries against eligibility criteria. Through EMR-based searches, researchers can identify eligible populations and initiate automated outreach, significantly accelerating participant identification. Once enrolled, EMRs contribute to operational efficiency by minimizing redundant assessments and enabling the seamless tracking of clinical endpoints.

However, these advantages can only be fully realized when key stakeholders—including trial sponsors—actively invest in supporting the necessary infrastructure and workflows. Despite growing interest in integrating EMRs into clinical trial workflows, the financial and strategic priorities of sponsors remain a critical determinant of implementation success. Sponsors traditionally fund protocol-driven procedures such as imaging or laboratory testing but often exclude infrastructure-related activities such as EMR integration, data extraction, and pre-screening personnel costs from trial budgets. One study highlights how these omissions lead to significant site-level burdens, with manual screening for early-phase trials costing over USD 300 per enrolled patient and requiring up to nine hours of staff time—resources that are rarely reimbursed [68].

Furthermore, the lack of standardized support systems exacerbates this divide. CTMS and EMRs are often underutilized due to limited interoperability and insufficient sponsor engagement in technical alignment [67]. While technological solutions exist, their effectiveness depends on early sponsor involvement in system design and workflow integration.

The application of EMRs in clinical trials has evolved beyond basic data storage or retrieval. Recent frameworks highlight that EMRs can serve a variety of research functions, including feasibility assessments, hypothesis generation, safety surveillance, performance monitoring, and patient recruitment. They are also increasingly used in pragmatic trials, comparative effectiveness studies, and even registry-based randomized trials. This expanding role reflects the growing recognition of EMRs as a versatile research tool capable of supporting both observational and interventional studies. Categorizing EMR use based on trial function—such as eligibility screening, endpoint ascertainment, or healthcare resource evaluation—offers a structured approach to their integration and may guide researchers in selecting appropriate EMR functionalities for specific study designs.

Beyond their capacity to streamline workflow and improve data accuracy, EMRs offer operational advantages that can directly enhance trial efficiency. Through the reuse of data originally collected during routine care, EMRs reduce redundant data entry and minimize transcription errors commonly associated with manual processes [69]. They also enable real-time or near-real-time access to clinical information, supporting rapid decision making and improving patient safety through timely monitoring. In addition, EMRs can be leveraged for remote data monitoring and integration with digital tools, helping to reduce trial costs and expand accessibility across geographically diverse sites. These features position EMRs as more than a data repository—they act as a dynamic infrastructure that supports both the conduct and scalability of modern clinical trials.

**Figure 3:**
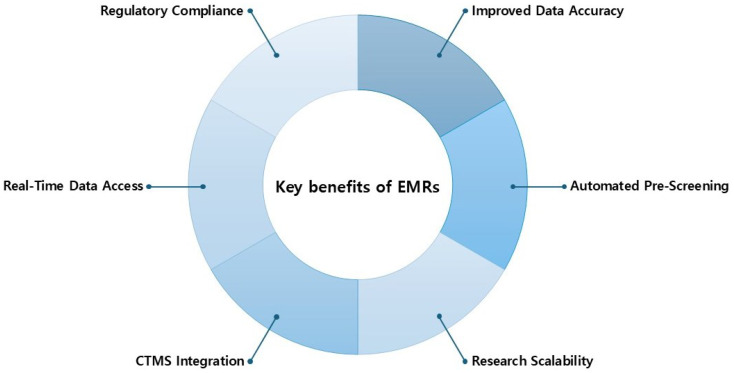
Key benefits of EMRs.

The primary benefits associated with EMR integration in clinical research are presented in Figure 3.

## 4. Expanding Applications of EMRs in Research

### 4.1. Real-World Data and Trial Design Innovations

EMRs are increasingly recognized as essential tools in the design and execution of clinical trials. As a key source of real-world data (RWD), EMRs help address limitations in traditional randomized controlled trials (RCTs), such as high costs, limited generalizability, and logistical burden. Regulatory agencies including the FDA and EMA now accept real-world evidence (RWE)—often derived from EMRs—for medical product approvals, further validating their role in modern research infrastructure [70,71].

EMRs enable a range of innovative trial designs, such as cluster randomized and stepped wedge designs, which improve trial efficiency and reduce ethical concerns. They also support the creation of external control arms (ECAs) by supplementing or replacing placebo groups with retrospective clinical data. In idiopathic pulmonary fibrosis (IPF) research, EMR-derived ECAs reduced trial duration, although shifts in treatment patterns over time introduced challenges [72].

Incorporating EMRs into clinical trials also enhances real-time patient monitoring and stratified recruitment. Integration with artificial intelligence (AI) allows the automated selection of high-risk cohorts and tracking of composite endpoints like hospitalization and survival. For example, Apple Watch ECG data were synchronized with EMRs in a Mayo Clinic study to enable real-time clinical monitoring, demonstrating the potential for wearable-linked EMR applications [73].

EMRs streamline the recruitment process by automating eligibility screening and facilitating digital phenotyping. The ADAPTABLE trial demonstrated the value of linking EMRs with claims data to identify eligible participants. AI-based NLP further enables refined cohort definitions using unstructured clinical notes and test results.

In genomics-informed trial designs, EMRs are used to apply polygenic risk scores (PRSs) and other Genome-Informed Risk Assessment (GIRA) frameworks. These approaches integrate clinical variables such as BMI, blood glucose, and family history with genetic data to identify high-risk individuals and guide preventive strategies. This has been applied to diseases such as coronary artery disease and breast cancer [74]. However, concerns remain regarding data bias, quality control, and clinician education.

At the system level, EMR infrastructure can enable cross-institutional collaboration. The IntegrIT project in Sweden utilized a national Health Information Exchange (HIE) platform to link disparate EMR systems, allowing access to trial documents including informed consent forms and protocol updates. This framework streamlined participant recruitment and communication across sites [75].

EMRs have also been applied to population health research and disease-specific interventions. For chronic kidney disease (CKD), EMRs support patient identification, risk stratification, and clinical decision support (CDS), enabling scalable management across large populations [76,77]. In primary care, EMR-based tools have increased screening and detection rates for intimate partner violence (IPV) through non-intrusive alerts, self-administered questionnaires, and CDS templates. However, disparities in screening completion and clinician burden remain challenges [78].

While the methodological potential of EMRs in generating RWE is increasingly recognized, the broader adoption of such data also hinges on evolving legal and policy frameworks. The secondary use of EMRs holds significant potential for advancing clinical research and public health monitoring. Regulatory agencies are beginning to recognize that standardization and governance of EMR data are essential for realizing these benefits. For instance, the AMIA white paper emphasizes the urgent need for a national framework that supports secure, ethical, and technically sound secondary use of health data through well-defined policies, data standards, and stakeholder collaboration [79]. Without such a framework, fragmented data governance and inconsistent technical practices inhibit effective data reuse across institutions and jurisdictions.

U.S. federal law—particularly the HIPAA Privacy Rule—permits certain secondary uses of patient data (e.g., public health surveillance, quality improvement) under specific conditions, while also acknowledging the growing complexity of electronic health information (EHI) ecosystems. Ramanathan et al. note that legal infrastructures must evolve in parallel with health IT systems, pointing to data use agreements (DUAs) and inter-organizational MOUs as key legal tools that enable compliant and secure secondary use [80]. These tools are especially vital for the cross-jurisdictional sharing of EMR data, such as in multicenter clinical trials or public health responses.

The strategic alignment of regulatory guidance across agencies—such as health IT standard-setters (e.g., ONC, HL7) and trial oversight bodies (e.g., FDA)—would not only enhance data interoperability but also build trust among sponsors, providers, and patients. As technical and legal infrastructures continue to mature, coordinated efforts to promote standardized, privacy-conscious EMR reuse could substantially accelerate the integration of RWD into clinical development and policy evaluation.

Beyond streamlining workflows in clinical trials, EMRs are increasingly recognized as a key source of RWD. When systematically collected and analyzed, these data can be transformed into RWE that supports not only clinical trial endpoints but also broader decisions in regulatory approval, reimbursement evaluation, and healthcare planning [71].

Previous studies have shown that RWD from EMRs can address a wide range of research questions extending beyond efficacy to include safety monitoring, long-term treatment outcomes, patient subgroup responses, and the utilization of healthcare resources. To enhance the relevance and acceptability of this evidence, a structured approach has been proposed in which the type of clinical or policy question is deliberately matched with the most suitable data source [81].

For example, EMRs may be more appropriate for questions involving clinical practice patterns or patient trajectories, while claims data may be preferred for economic analyses. Applying this question-driven framework within the design of the trial can help ensure that the evidence integrated with EMRs is methodologically sound and aligned with the expectations of decision-making bodies [82].

The use of EMRs in clinical trials (EMR-CTs) is gaining momentum as a cost-efficient alternative to traditional randomized controlled trials (RCTs). EMRs offer practical advantages in identifying study candidates, facilitating follow-up, and enabling larger-scale recruitment across diverse populations. Their scalability and real-time accessibility make them well suited for pragmatic trial designs embedded within learning health systems, offering more generalizable insights into clinical effectiveness under real-world conditions.

Beyond feasibility, there is growing discussion about the scientific validity of EMR-CTs. Critics highlight concerns such as selection bias, missingness, poor standardization of outcome measures, and difficulties in drawing causal inferences from EMR data due to inherent data limitations. Nonetheless, frameworks such as the Structured Pre-Approval and Post-Approval Comparative study design framework to generate valid and transparent real-world Evidence (SPACE) and the SPIFD tool have been developed to guide EMR-CTs toward regulatory-grade quality [70].

Importantly, EMR-CTs are not monolithic; their methodological designs range from conventional RCTs utilizing EMRs to more pragmatic designs such as cluster randomized trials or stepped wedge trials. For instance, cluster randomized trials are often selected to mitigate contamination and enhance administrative feasibility, while stepped wedge designs address ethical concerns by eventually delivering interventions to all participants. These approaches enhance real-world applicability while still preserving elements of randomization.

A key insight is that EMR-based designs must be carefully aligned with research objectives. As Franklin and Schneeweiss argue, RWD analyses may substitute for RCTs in hypothesis-evaluating treatment effectiveness (HETE) questions under specific conditions—particularly when data quality is high, exposure is well defined, and confounding can be adequately addressed. This underscores the importance of methodological rigor in ensuring internal validity, even when trials are embedded in routine care settings.

In practice, embedding randomization within health system networks—using tools like digital phenotyping, automated recruitment via patient portals, and structured eligibility queries—has been shown to accelerate enrollment while preserving generalizability. While challenges remain, notably in consent acquisition, data harmonization, and ethical oversight across multiple sites, regulatory bodies such as the FDA and EMA now actively endorse RWE, further legitimizing EMR-CTs as a viable research modality [70].

Thus, EMR-CTs are transitioning from logistical convenience to a potentially rigorous and policy-relevant mode of clinical investigation. Their value lies not only in cost and speed but also in enabling a new evidence paradigm that better reflects everyday clinical settings and heterogeneous patient populations.

### 4.2. Artificial Intelligence, Automation, and EMR Usability

The integration of AI and machine learning (ML) technologies into EMRs has significantly expanded the analytical capabilities of clinical research. These technologies support a range of trial-related activities, from patient stratification and recruitment to outcome prediction and safety monitoring, enhancing both the efficiency and precision of trial workflows.

ML models are increasingly used to improve diagnostic accuracy and reduce healthcare costs. For example, models have been applied to predict oxygen demand in COVID-19 patients using vitals, radiographs, and lab data. Deep learning has also supported the classification of skin cancer, diabetic retinopathy, and respiratory distress in older adults.

NLP, a subfield of AI, enables the interpretation of unstructured EMR data such as clinical notes. NLP has proven especially effective in the early detection of conditions like dementia by extracting risk patterns from patient narratives.

Causal machine learning (CML) enables individualized treatment effect estimation based on EMR data. These models account for heterogeneity in treatment responses across patient subgroups, advancing the vision of precision medicine [83].

EMR-integrated AI tools also enhance clinical trial screening. In gynecologic oncology, AI-enabled patient matching has increased enrollment efficiency by over 50% in some centers [68]. Likewise, a neural network model developed using VA data was able to predict acute kidney injury (AKI) risk 48 hours in advance [84].

Commercial tools such as IBM Watson illustrate the potential of AI to synthesize EMR content with the medical literature, enabling accurate predictions of readmissions and treatment outcomes in complex populations such as diabetes and mental health disorders [85].

Electronic form entry errors remain a persistent barrier to the optimal use of EMRs. These errors include missing required fields, inappropriate default selections, and misclicked dropdowns—issues that compromise data quality and patient safety. A study by Zandieh et al. identified that incomplete or incorrect electronic documentation was among the most commonly reported problems during EMR implementation, often resulting in workflow disruption and provider frustration [39].

Similarly, a large-scale scoping review highlighted that poor interface design and rigid form structures contributed to user fatigue, which in turn increased the likelihood of entry errors and reduced the reliability of captured data.

These usability issues are not trivial; they can lead to clinical decision errors, billing inaccuracies, and undermined trust in EMR-derived research data. To address these challenges, researchers have proposed integrating human factor engineering into form design and adopting real-time validation mechanisms that alert users before the submission of faulty entries. Redesigning EMR interfaces to be more intuitive and adaptive is essential for reducing documentation burden and ensuring accurate data capture [37].

**Figure 4:**
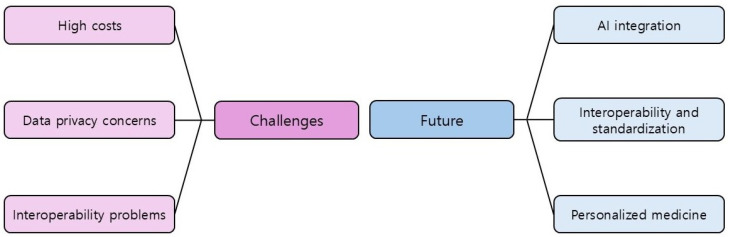
Challenges and future of EMRs in clinical trials.

The main challenges and future directions in EMR adoption are mapped in Figure 4.

## 5. Operational and Strategic Challenges in EMR-Driven Clinical Trials

Despite the expanding role of EMRs in modern clinical research, their integration into the trial infrastructure continues to face operational and strategic challenges. These issues—spanning data quality, system heterogeneity, sponsor engagement, and regulatory governance—limit the scalability and consistency of EMR-enabled clinical trials.

Among these, concerns about data reliability and completeness remain foundational barriers to EMR use in research. Many EMRs were originally designed for administrative or billing purposes, not research, raising concerns about the accuracy, consistency, and completeness of their data entries [86]. Variability in coding practices and incomplete documentation can introduce bias and threaten outcome validity [87]. These problems are exacerbated in systems where patients receive care across unlinked facilities, limiting longitudinal data access and reducing the utility of EMRs in trials that require continuous follow-up or comprehensive endpoint tracking [88]. Timely access is another critical issue: diagnoses and discharge summaries may be entered weeks after patient encounters, impeding real-time patient screening, safety monitoring, and endpoint adjudication in time-sensitive studies [89].

Beyond data quality, structural incompatibilities between EMR systems also complicate multi-site and international trial efforts. Differences in data schemas, field definitions, and software architectures across EMR platforms complicate harmonization and large-scale data aggregation, especially in multi-site or international trials [90]. These technical disparities are further compounded by regulatory and legal fragmentation. Jurisdictions differ widely in their requirements for data sharing, informed consent, and secondary use of EMR data, making global implementation logistically and ethically challenging [82,91].

These technical and regulatory hurdles are further compounded by misaligned incentives among key stakeholders—particularly trial sponsors. Sponsors typically fund protocol-driven procedures such as laboratory tests or imaging but often exclude infrastructure costs—such as EMR integration, automated screening, or data extraction—from site budgets. These omissions place a substantial burden on trial sites, especially in resource-constrained or community settings, where manual screening may cost over USD 300 per enrolled patient and require hours of staff time [68]. Furthermore, CTMS and EMR platforms remain underutilized due to poor interoperability and limited sponsor engagement during the design phase [67]. While regulatory agencies like the FDA are increasingly accepting RWE from EMRs in drug approvals [92], sponsors have been slow to recognize the strategic value of EMR-enabled trial models.

At the same time, privacy and governance concerns continue to pose significant ethical and operational risks. Even when data are deidentified, risks of reidentification or unauthorized access persist. Regulatory frameworks such as the Common Rule and HIPAA provide important guardrails, but as health IT systems evolve, continuous adaptation is needed [80,93]. Cross-institutional data sharing further necessitates legal tools like data use agreements (DUAs) and memoranda of understanding (MOU) to ensure compliant and secure data exchange.

Even with technical and legal safeguards in place, the absence of a coordinated operational model remains the most systemic barrier. Most EMR-to-trial workflows are site-specific and ad hoc, lacking standardized protocols for data quality, traceability, and accountability. Without coordinated efforts to align technical protocols, governance models, and evaluation metrics, the broader vision of EMR-integrated clinical research remains fragmented. Establishing such frameworks will be essential not only for technical integration, but also for broader stakeholder trust and long-term sustainability.

Related to concerns about data quality, electronic form-based data entries remain susceptible to human and system-level errors. A recent quality improvement study at a major Canadian cancer center reviewed 776 transferred EMRs and found that 15.3% of patient charts contained at least one documentation error, the majority (85.9%) of which were classified as major errors that could affect clinical care [94]. These included discrepancies in cancer diagnosis, staging, and treatment plans, often resulting from manual input mistakes, inconsistent formatting, or the propagation of outdated information through copy–paste practices. For example, incorrect Gleason scores in prostate cancer patients were found to be repeatedly propagated across visits, potentially impacting treatment decisions.

These findings underscore the risk of error propagation in digital systems, especially in high-complexity cases or institutions using multiple EMR systems. Improving data integrity will require a combination of automated validation rules, standardized documentation protocols, and interface designs that reduce cognitive load and entry ambiguity. Importantly, error mitigation strategies must extend beyond technical fixes to include adequate user training and quality control workflows during data transfer or migration events.

## 6. Strategic Guidelines for EMR Integration in Clinical Trials

Effectively leveraging EMRs in clinical trials requires more than isolated technical fixes—it demands a comprehensive, cross-sector strategy that addresses persistent barriers while laying the foundation for future innovation.

First and foremost, foundational issues of data quality and interoperability must be addressed. Ensuring accuracy, completeness, and consistency in EMR data requires structured data entry formats, automated validation protocols, and standardized clinical coding systems. Widespread adoption of interoperable data standards such as HL7 and FHIR will allow for more seamless data sharing between institutions, supporting multi-site trials and reducing redundancy. HIEs and blockchain-supported data architectures can further enhance the security and accessibility of patient information, enabling decentralized research models and longitudinal tracking of clinical endpoints. Recent proposals for eSource Record systems underscore the value of collecting validated clinical data directly from EMRs, offering sponsors and regulators greater confidence in its traceability and methodological rigor [95].

Closely tied to data infrastructure are ethical and legal frameworks that govern the secondary use of EMR data in research. Compliance with established policies such as HIPAA, GDPR, and the Common Rule is critical for protecting patient privacy and maintaining public trust. Strategies such as data deidentification, transparent informed consent procedures, and role-based access permissions help mitigate risks of reidentification and unauthorized access. As EMR use expands across international jurisdictions, legal tools like data use agreements (DUAs) and memoranda of understanding (MOU) will become essential for enabling compliant cross-institutional data exchange [80]. To foster collective learning and reduce duplication, some have proposed the creation of open access repositories documenting real-world use cases of EMR-enabled trials. Such repositories could help clarify what constitutes acceptable EMR-based evidence, standardize expectations across stakeholders, and support regulatory decision-making [82,96].

Technological innovation will also play a central role in unlocking the full value of EMRs. Artificial intelligence and machine learning models can be used to analyze large-scale EMR datasets, assist in trial monitoring, and develop predictive models for treatment outcomes. NLP extends these capabilities to unstructured data, enabling automated cohort identification and digital phenotyping. However, these tools must be supported by user-centered system design. Poor interface usability and rigid documentation structures contribute to provider fatigue, documentation errors, and loss of data fidelity. Integrating human factors engineering into EMR design—through intuitive interfaces, automated error alerts, and targeted training—can reduce these burdens while improving data quality and clinical workflow efficiency.

Equally important is aligning stakeholders—especially sponsors, regulators, developers, and investigators—around shared operational frameworks. Sponsors must expand their role beyond funding protocol-specific procedures and invest in the underlying EMR infrastructure that enables pre-screening, real-time data access, and secure information exchange. Studies have shown that current funding gaps in infrastructure often leave trial sites to absorb the cost of EMR integration, particularly in community or under-resourced settings [68]. Improving this situation will require early sponsor engagement in technical alignment, joint platform development, and budgetary inclusion of EMR-related costs. Furthermore, coordinated pre-trial planning among stakeholders is essential to ensure the consistent implementation of governance policies and data standards, as well as adequate personnel training [69].

Ultimately, optimizing EMR integration in clinical trials demands more than patchwork fixes—it requires long-term strategies rooted in technical soundness, ethical accountability, stakeholder collaboration, and design-driven usability. By advancing these efforts collectively, EMRs can become not only administrative tools but foundational platforms for accelerating research, enhancing equity, and reshaping the future of clinical trial design.

**Figure 5:**
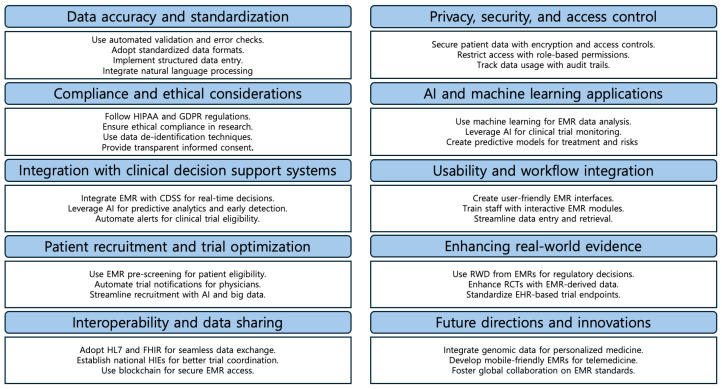
Key guidelines for optimizing electronic medical records in clinical trials.

The simplified workflow for the above guidelines are illustrated in Figure 5.

## 7. Limitations

This review was conducted using a narrative and integrative approach, synthesizing literature from clinical research, health informatics, and policy studies to explore the evolving role of EMRs in clinical trials. While this method enabled broad conceptual coverage, it lacks the formal reproducibility and protocol structure of systematic reviews. No standardized method (e.g., PRISMA) was applied, and selection was based on thematic relevance, which may introduce selection bias.

Additionally, although this review includes case examples from multiple healthcare systems, it draws primarily from U.S.-based studies and infrastructures. This reflects the historical prominence of U.S. institutions in EMR development and clinical research digitization. However, it also limits the generalizability of certain observations. Future reviews may benefit from a more systematic, geographically balanced study distribution to capture global practices and innovations.

## 8. Conclusions

Electronic medical records (EMRs) have emerged as a foundational infrastructure for modern clinical trials, offering substantial benefits in data accuracy, operational efficiency, and research scalability. This review has traced the evolution of EMRs from administrative tools to dynamic platforms for trial execution, examined their integration into clinical research workflows, and assessed the technological, ethical, and organizational barriers that limit broader adoption.

While EMRs are increasingly used for participant recruitment, real-world data generation, and decision support, persistent challenges in data quality, system interoperability, stakeholder coordination, and privacy governance remain. These barriers constrain the consistency and generalizability of EMR-enabled trials across institutions and countries. This review has emphasized the need for stronger regulatory alignment, financial support from sponsors, and cross-sector planning to overcome these limitations.

To support the sustainable and equitable integration of EMRs into trial infrastructure, this review has also proposed strategic guidelines focused on four key areas: data quality and interoperability, ethical and legal frameworks, technological innovation, and stakeholder collaboration. Realizing this vision will require not only technical improvements but also institutional commitment, transparent data governance, and coordinated investment across the clinical research ecosystem.

By aligning technical standards, reinforcing privacy protections, and promoting collaborative operational models, EMRs can transition from passive data repositories to active engines of innovation. As healthcare systems increasingly prioritize personalized care and evidence-based policy, EMRs are uniquely positioned to bridge clinical care with research—advancing not just trial efficiency but also the broader goals of health equity and knowledge generation.

## Data Availability

The data that are discussed in this article are presented in cited studies.
